# Editorial

**Published:** 1871

**Authors:** 


### Editorial.

***Rush Medical College.***

The Twenty-Ninth Annual Session of Rush Medical College opened on the 27th ult. The usual introductory exercises took place in the lower lecture room of the college, in the evening, and the regular course of lectures commenced the next morning at nine o'clock. Prof. H. M. Lyman pronounced the Introductory Lecture, which we shall give our readers, by his permission, in the next number of the JOURNAL, and therefore shall not anticipate opinions by eulogizing it.

Professor Freer, again returned from his annual European trip, and we are happy to say in splendid health and spirits, gave the first regular lecture of the course, and was followed by each member of the Faculty in the order of the printed programme.

The only changes we have to note are the retirement of Prof. E. Ingals from the active duties of the chair of Materia Medica and Medical Jurisprudence, his appointment by the Board of Trustees to the Emeritus Professorship of the same branches, and the election and installation of J. H. Etheridge, M.D., an alumnus of the college, in the vacated position. The Board of Trustees accompanied their election of Prof. Ingals to the Emeritus Professorship by a warm expression of their high sense of the ability with which he had discharged the duties of his collegiate position, their profound respect for his personal character, and best wishes for his future prosperity and happiness.

Prof. Etheridge, the new incumbent, advances to the position with the prestige of thorough scholarship, the diploma of Rush, foreign travel and study, considerable experience as a lecturer, personal culture and refinement, and the absolute knowledge on the part of the individual members of the Faculty of his eminent qualifications for the chair.

The senior editor of this JOURNAL is happy to announce that WALTER HAY, M.D., his associate, has kindly consented to deliver, in connection with the course on Practical Medicine, a series of lectures on the Pathology and Therapeutics of the Brain and Nervous System. Due notice will be given of the time when these lectures will occur, and the senior editor takes the liberty, hereby, of inviting the profession to attend them at their convenience, and he does not hesitate to say they will find themselves amply repaid for the time occupied.

The class assembled is the largest which has been present at the opening of the course for several years.

Surrounded as the college is by a cordon of competing schools, the most of which throw open their doors "without money and without price," the Faculty had every reason to anticipate a considerable falling off in attendance the present session, and are in fact surprised, and of course gratified, at the large attendance. The great book-house of W. B. Keen, Cooke & Co. (our publishers) inform us that their sales of medical books surpass those at the opening of any previous session.

The senior editor congratulates the friends of "Old Rush" on its flattering condition and prospects, and attributes them mainly to its teachings of sound doctrine—of physiological medicine, surgery and obstetrics—conservative in all branches.

### *Calamity.*

The foregoing paragraphs were written for the October No. of the JOURNAL. That No. was delayed a few days for the purpose of announcing the initial exercises of Rush Medical College. The edition was bound and ready to mail to subscribers. The reason why it was not mailed is known to all the world.

There was a fire in Chicago, on Sunday night and Monday morning, Oct. 8th and 9th.

That fire burned over an area in the city of 2,124 acres.

It burned 17,450 buildings, and besides public and business edifices, it consumed the homes of 98,500 people. $300,000,000 were destroyed in a few hours.

Writers of all varieties have exhausted language in description of this supreme catastrophe. The secular prints have been full of it—books have been written about it. We have no words for anything more than the simple facts, above given, and the plain statement that in the holocaust the immense establishment of our Publishers, Messrs. W. B. Keen, Cooke & Co., was totally destroyed. With it went the mail book of the JOURNAL, and the back Nos. for the last two years. Compared with the destruction of their vast stock of books and stationery, this was a comparatively trivial loss, but it was an exceedingly serious one to the JOURNAL itself.

Messrs. Rand, McNally & Co., Printers of the JOURNAL, were equally unfortunate. They were perhaps the leading house of the Northwest, and the array of their presses, machinery, and supplies of every sort, was utterly bewildering to everybody, except printers of the largest experience. They saved not a pound of type, not a press, a hammer, or a sheet of paper.

As before stated, the October No. of the JOURNAL was burned with the rest of their stock. Not a single No. was saved. Accidentally, one of the workmen had wrapped a photograph in the proof sheet of the form which contained the editorial above. The photograph and its wrapper were in Ohio, and when the proof sheet came back to us, long after the fire, it looked to us literally like "a brand plucked from the burning."

With characteristic energy, Messrs. Keen & Cooke not only made immediate arrangements for re-establishing their general business, but also for the continuance of the JOURNAL. We had hoped for a delay not beyond a month at the latest. But circumstances which we could not foresee prevented. Our Printers have nearly reorganized their immense establishment, but, in consequence of the non-reception of material, which had to be manufactured to order, delay has been inevitable. If we had anticipated this, we should have sent the JOURNAL elsewhere, for temporary printing.

We give you here a consolidated No. It may be called a Fire Number, if desired.

Being now supplied with the usual facilities, subsequent Nos. of the JOURNAL will be issued regularly, as in the time before the fire.

RUSH MEDICAL COLLEGE

Lost its building, and everything it contained. Not a scrap of paper, a bit of apparatus, a book or a bone, was left. Prof. Lyman, from the top of a building, several miles away, saw, through a telescope, the massive self-sustaining roof fall, with a grand rush of flame heavenward succeeding, and was thus the last man to look upon the erect building, and probably the only one who saw it burn, for, while it was being consumed, around for blocks, (we were about to write for miles) there was but a roaring sea—a hell of flame.

Profs. Freer, Blaney, Powell, Holmes, had their homes and offices and other property utterly burned up. Prof. Gunn lost his library, instruments, office furniture, etc. Prof. Rea lost his office, and a large sum in buildings, etc. Drs. Parkes and Wadsworth also lost everything. But space and time prevent details. In another place, in the JOURNAL, will be found an appeal to the profession on behalf of the College, by the Alumni, with a paper, also, from the Board of Trustees, to each of which the earnest attention of readers is solicited.

After an interim of *four* days, only, the lecture course was resumed, and has since been in regular progress. The authorities of Cook County Hospital kindly granted the use of the amphitheatre of their building, for the remainder of the session.

It is gratifying to state that, although nearly every one of the students was burned out, and some lost their all, yet a majority of the class returned to continue their attendance. We do not hesitate to say that, notwithstanding everything, the session thus far has proved pleasant and useful to all concerned.

Many of the Medical Colleges throughout the country tendered free tickets to all students who paid for them here—the Chicago Medical College being the first to make the offer. For this generosity they are entitled to the thanks of Rush Medical College. May they be spared an opportunity of our reciprocating the courtesy.

The rebuilding of the College, of course, is determined upon. It will be finished for occupancy before the next session.

***St. Luke's Hospital.***

M. O. Heydock, M.D., has been appointed by the Board of Trustees of St. Luke's Hospital, Attending Physician, and I. N. Danforth, M.D., Pathologist.

***The American Practitioner***

Lays us under renewed obligations by sending the two volumes of their issue for 1871, beautifully bound. It is a very valuable periodical, and we cheerfully commend it to our own subscribers, who wish (and who does not?) to take more than one journal. Address the Publishers, Louisville, Ky., and enclose $3.00.

**Bromo-Chloralum.**

This is a very efficient antiseptic, disinfectant, and deodorizer. It possesses the decided advantage of being in itself inodorous and nearly devoid of irritant properties, when locally applied. The writer has used it in many instances as a substitute for solutions of carbolic acid, with very pleasant results. It is not anæsthetic or sedative, like the latter, in its local application, and hence, in many instances, is preferable. We consider it a valuable addition to our medical armamentarium.

**Diaries.**

Our thanks are due to S. W. Butler, M.D., Editor of the Philadelphia Reporter, for a copy of his very excellent "*Physician's Daily Pocket Record*," comprising a Visiting, many useful Memoranda Tables, etc. This book is suitable for any year, and in some particulars is the most convenient issued.

Lindsay & Blakiston issue their diary for 1872 in the form now well known by the profession, it having been many years the same.

**A Query to the Board of Health.**

Some of your employes take pains to urge the inmates of houses where small pox is present to wear bags of camphor and assafœtida as preventative. Is this by official order?

---

CHICAGO, November 1st, 1871.

*To Whom it May Concern:*

This is to certify, that the resident Alumni of Rush Medical College, of the City of Chicago, at a meeting held at the house of E. Ingals, M.D., on the eve of October 17, 1871, appointed the following Alumni an Executive Committee, to draft and present an appeal to the Alumni and friends of the College, for aid to rebuild and refurnish the College Building, viz: T. D. Fitch, M.D., Chairman; H. A. Johnson, M.D., V. L. Hurlbut, M.D., C. T. Parkes, M.D., Ben C. Miller, M.D., and F. A. Emmons, M.D.

E. INGALS, M.D., *Chairman.*

CURTIS T. FENN, M.D., *Secretary.*

